# Role of endoscopic ultrasound in diagnosis and management of autoimmune pancreatitis

**DOI:** 10.1007/s12664-025-01852-x

**Published:** 2025-09-25

**Authors:** Gauri Kumbhar, Akhil Mahajan, Rahul Puri, Sridhar Sundaram

**Affiliations:** Division of Interventional Endoscopy, Department of Digestive Diseases and Clinical Nutrition, Tata Memorial Hospital, Homi Bhabha National Institute, Dr. E Borges Road, Parel, Mumbai, 400 012 India

**Keywords:** Autoimmune pancreatitis, Endoscopic ultrasound, Pancreas

## Abstract

Autoimmune pancreatitis (AIP) is a rare inflammatory disorder of the pancreas, often mistaken for pancreatic cancer. Despite definitive diagnostic criteria such as histology, imaging, serology, other organ involvement and response to therapy (HISORt), International Consensus Diagnostic Criteria (ICDC) and Japan Pancreas Society (JPS) criteria being available for AIP, making a diagnosis of AIP remain a rarity in the evaluation of a pancreatic mass or distal bile duct obstruction. A significant proportion of patients are diagnosed only after surgical resection. Endoscopic ultrasound (EUS) is the main modality for establishing diagnosis of AIP. Considering the rarity of the disease, there are no classical findings on EUS associated with AIP. Histopathology remains the crux for diagnosing AIP with need for EUS-guided sampling. Both focal and diffuse forms of AIP are described with different EUS findings in both. The focal form mimics pancreatic cancer closely. The disease is also known to have extra-pancreatic involvement with cholangiopathy also seen often in association. Diffuse involvement of the pancreas is unusual and may be rarely seen with diffuse pancreatic infiltrative diseases. The primary consideration remains differentiating AIP from carcinoma of pancreas, where EUS plays a significant role. Adjunct techniques such as EUS-guided elastography and contrast harmonic EUS can add value in diagnosing AIP. Advances in tissue sampling, including availability of better needles for core biopsy, have aided in improving the diagnostic yield of AIP. In this narrative review, we aim to highlight the increasing role of EUS for establishing diagnosis of AIP while elaborating its role in evaluation, sampling and therapeutic monitoring.

## Introduction

Autoimmune pancreatitis (AIP) is an immune-mediated chronic fibroinflammatory condition of the pancreas that typically responds well to corticosteroid therapy [1]. It is a subset of chronic pancreatitis that has unique histologic, morphologic and clinical characteristics [2]. It has been reported to constitute up to 2% of all chronic pancreatitis cases, with a prevalence of less than one per 100,000 people [3]. Over the past few years, the global prevalence of AIP has risen as a result of improved characterization and increased awareness of the disease [4, 5]. In a retrospective case series from a tertiary centre in north India, Noor et al. reported that autoimmune pancreatitis accounted for approximately 2.6% of chronic pancreatitis cases evaluated over a three-year period, highlighting the need for a high index of suspicion in diagnosing autoimmune pancreatitis [6].

According to The International Consensus Diagnostic Criteria (ICDC), there are two sub-types of AIP. Type-1 AIP (AIP1), also known as lymphoplasmacytic sclerosing pancreatitis, is the pancreatic manifestation of IgG4-related systemic disease. It is marked by high serum IgG4 and IgG4-rich lymphoplasmacytic infiltration with sclerosis [7]. While elevated serum IgG4 levels are a hallmark of Type-1 AIP, Indian studies have reported variability. In a north Indian cohort, Rana et al. noted that nearly 20% of patients with Type-1 AIP had normal IgG4 levels, indicating that reliance on this parameter alone may reduce diagnostic sensitivity in the Indian population [8]. In a large series from western India by Jain et al. [9], IgG4 was normal in 32% patients with IgG4-related digestive disease.

The disease commonly affects multiple organs, primarily the pancreatic-hepatobiliary system (45%), salivary glands (37%) and other organs. Predominantly seen in Asia, it typically presents with obstructive jaundice in elderly males and can be diagnosed without pancreatic biopsy when other diagnostic criteria are met [10]. In a previous study by Jain et al. [9], other organ involvement was seen in 18% patients with IgG4-related digestive disease. In contrast, Type-2 AIP (AIP2) features a distinctive histological pattern called idiopathic duct-centric pancreatitis with granulocytic epithelial lesions. More prevalent in Europe and the US, it typically affects younger patients in their 30s and 40s without gender predilection. Unlike AIP1, it presents primarily with abdominal pain and acute pancreatitis, remains confined to the pancreas and shows minimal to no IgG4 activity [11]. In a study by Rana et al. from PGIMER, north India, among 18 patients diagnosed with autoimmune pancreatitis, Type-1 AIP accounted for 72% (13 patients; four definite, nine probable), while Type-2 AIP was identified in only 11% (two patients). This highlights the predominance of Type-1 AIP in the Indian population, mirroring global trends [8].

A definitive diagnosis and differentiation into sub-types requires histological confirmation through tissue sampling [12]. Importantly, Type-1 and Type-2 AIP cannot be distinguished based on imaging, as both sub-types may present with either diffuse or focal pancreatic involvement [13]. While steroids effectively induce remission in over 95% of cases, disease relapse occurs in approximately 30% of Type-1 and 9% of Type-2 patients after initial remission [14]. Recently Type-3 AIP has also been described in patients receiving immune checkpoint inhibitor therapy for various cancers [15].

## Evolution of diagnostic criteria

The diagnostic framework for AIP has evolved significantly over time. Initially the diagnosis of AIP was based on the Japanese Consensus, originally discussed in 2002 with subsequent modifications done in 2006 and 2011 [11]. Klöppel et al. from the Mayo Clinic came up with the histology, imaging, serology, other organ involvement and response to therapy (HISORt criteria) for diagnosis of AIP in 2006 [12]. To reach an international consensus for diagnostic criteria, ICDC were then defined by various experts in Honolulu in 2011 [13]. The diagnosis of AIP requires the following criteria: (1) imaging findings (parenchymal and ductal), (2) serologic findings, (3) other organ involvement (OOI), (4) histologic findings, (5) response to steroid therapy [14]. The ICDC is associated with high sensitivity (~ 90%) and specificity (~ 100%) for diagnosis of AIP [15]. The criteria hold true for both focal and diffuse forms of AIP and hence remains an integral part of the diagnostic algorithm [16]. Table 1 summarizes the different diagnostic criteria for AIP.

**Table 1:** Comparison of diagnostic criteria for autoimmune pancreatitis

Feature	JPS [11]	ICDC [16]	HISORt [13]
Pancreatic imaging	Diffuse/segmental enlargement with irregular narrowing of MPD	Level 1: Typical imaging (diffuse enlargement, capsule-like rim); Level 2: Segmental changes	Imaging evidence of pancreatitis (CT/MRI/ERCP)
Serology	Elevated IgG4	Level 1: ≥ 2 × ULN; Level 2: < 2 × ULN but above normal	Elevated IgG4 level
Histopathology	Lymphoplasmacytic sclerosing pancreatitis (LPSP) features	Type 1 AIP: LPSP (storiform fibrosis, obliterative phlebitis, IgG4 + cells); Type 2: IDCP (granulocytic EP)	Pancreatic biopsy showing LPSP or steroid responsiveness
Other organ involvement (OOI)	Not mandatory	Level 1: Typical (e.g. sclerosing cholangitis, retroperitoneal fibrosis); Level 2: Probable OOI	OOI such as sclerosing cholangitis, sialadenitis
Response to steroids	Yes	Considered supportive (response within 2–4 weeks is diagnostic if other features are indeterminate)	Diagnostic if response is dramatic and other causes excluded
ERCP findings	Irregular narrowing of MPD	Level 1: Long, narrow MPD stricture without upstream dilatation	Pancreatogram showing irregular MPD narrowing
Type classification	Not applicable	Differentiates Type 1 (IgG4-related) and Type 2 (idiopathic duct-centric pancreatitis)	Focused on Type-1 AIP
Unique features	Focus on practicality in Japanese population;	Most comprehensive criteria with levels of evidence; Distinction between definitive and probable for both Type 1 and Type 2	First to formally recognize Type-2 AIP; Strong emphasis on histology

*HISORt* histology, imaging, serology, other organ involvement and response to therapy, *ERCP* endoscopic retrograde cholangiopancreatography, *MPD* main pancreatic duct, *ULN *upper limit of normal, *AIP* autoimmune pancreatitis, *IgG4* immunoglobulin G4, *IDCP* idiopathic duct-centric pancreatitis, *OOI* other organ involvement, *CT* computerised tomography, *MRI* magnetic resonance imaging, *EP *Granulocytic epithelial lesion associated pancreatitis, *ICDC *International Consensus Diagnostic Criteria, *JPS *Japan Pancreas Society Criteria

## Imaging in AIP

**Fig. 1:**
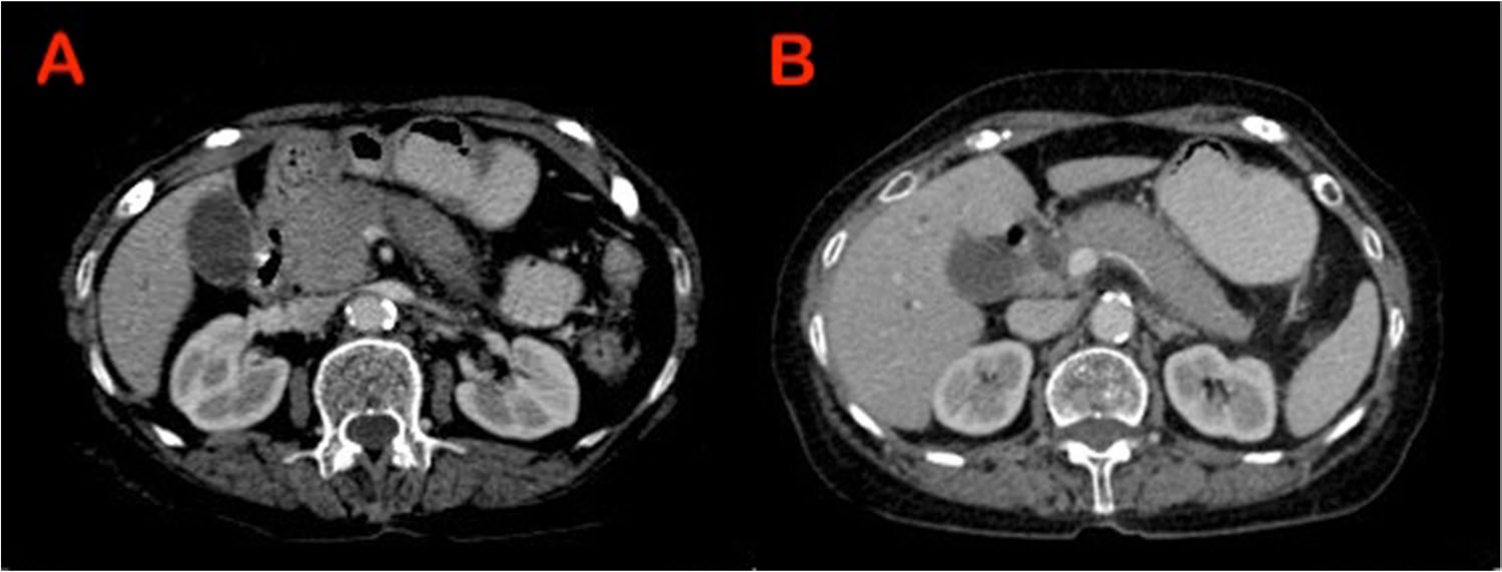
Computerised tomography (CT) showing sausage shaped enlargement of the (**A**) head of pancreas (**B**) Body and tail of pancreas - suggestive of diffuse autoimmune pancreatitis

**Fig. 2:**
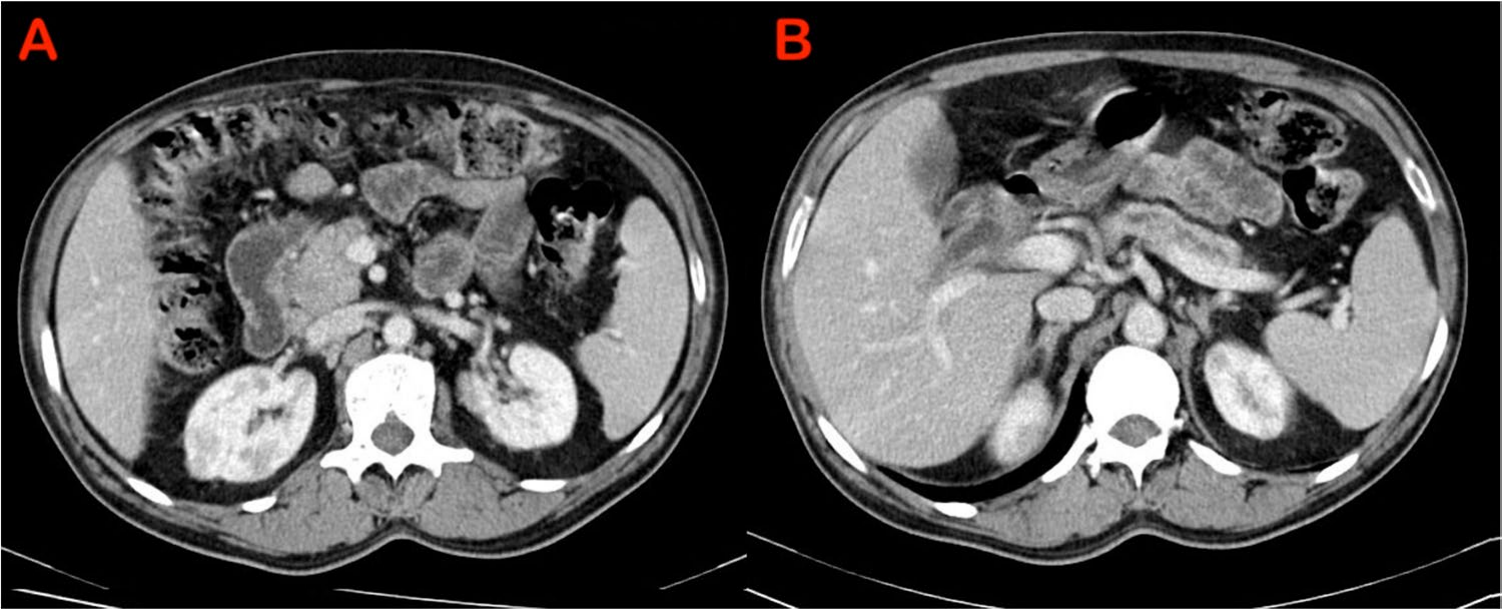
Computerised tomography (CT) shows (**A**) focal enlargement of the head of pancreas (**B**) mildly dilated pancreatic duct in body and tail - suggestive of focal autoimmune pancreatitis

**Fig. 3:**
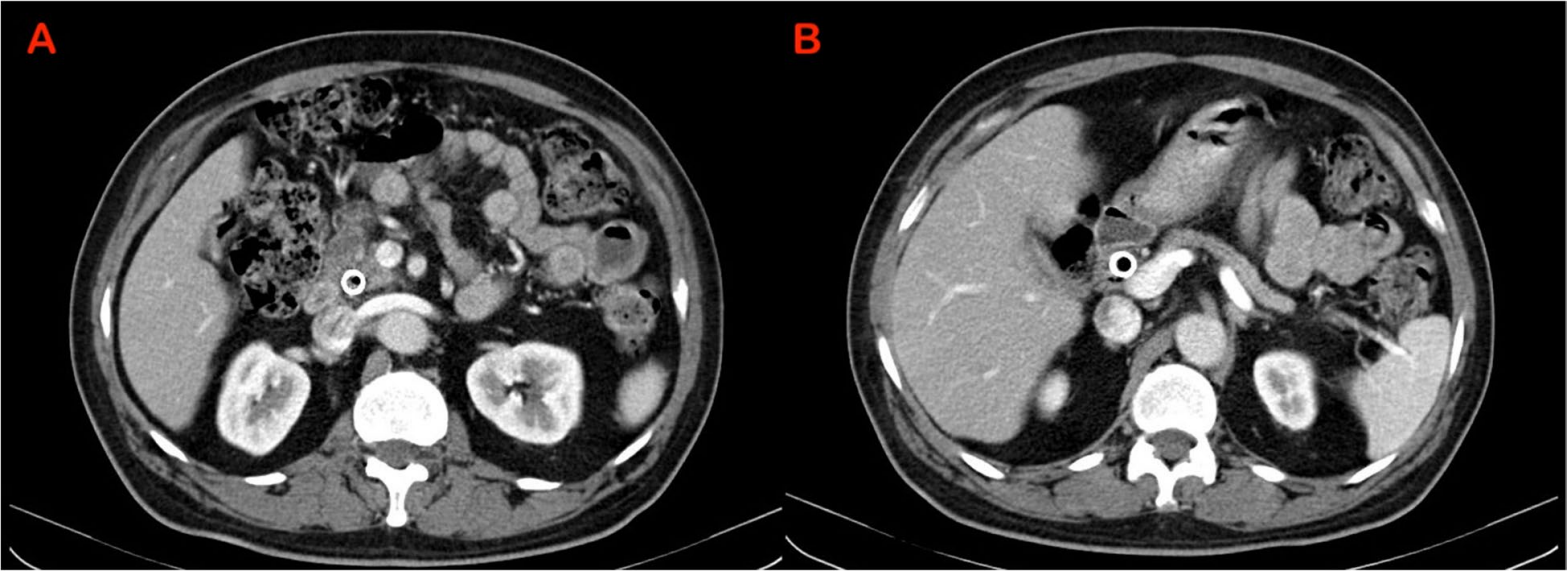
Computerised tomography (CT) shows post steroid treatment change in a patient with autoimmune pancreatitis (**A**) atrophy of the head of pancreas with a covered metal biliary stent in situ (**B**) atrophy of the body and tail of pancreas with undilated MPD

Characteristic findings of AIP on cross-sectional imaging include a diffusely or locally enlarged pancreas with distortion and/or loss of its lobular architecture, often described as a “sausage-shaped pancreas”. Additionally, a capsule-like rim and a distinctive delayed enhancement pattern are commonly observed. Pancreatic duct (PD) may show irregular narrowing (Figs. 1, 2, 3) [17, 18]. While the imaging features described in various diagnostic criteria for AIP, such as the ICDC, primarily rely on computed tomography (CT) and magnetic resonance imaging (MRI), the role of EUS in literature has been largely focused on obtaining tissue samples for diagnosis [16]. Notably, there are no established EUS-based diagnostic criteria for AIP. However, with recent advancements in EUS technology and techniques, there is a growing need to define standardized EUS criteria for AIP.

## Conventional EUS

EUS has emerged as a valuable diagnostic tool in the evaluation and management of AIP, owing to its high sensitivity, ability to assess both —pancreas and surrounding structures— and its role in guiding tissue sampling for histological analysis. There are no pathognomonic EUS imaging findings of AIP.

### Pancreatic findings

#### Diffuse AIP

**Fig. 4:**
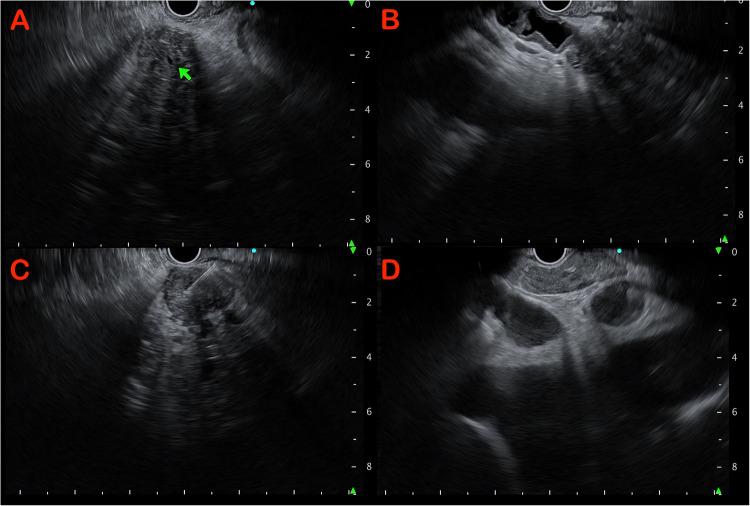
(**A**) Pancreatic head showing heterogenous echogenicity with undilated pancreatic duct. (**B**) Diffuse thickening of the bile duct seen on endoscopic ultrasound. (**C**) Biopsy taken from the pancreatic body which also appears heterogeneously hypoechoic. (**D**) Enlarged sub-carinal lymph node which was reactive seen in association with autoimmune pancreatitis

Diffuse pancreatic enlargement and altered echotexure is seen. Gland border is thickened and echogenic interlobular septa may be seen. There often is loss of normal salt and pepper pattern of the pancreas with bulky mildly hypoechoic parenchyma with hyperechoic strands seen within (Fig. 4). The MPD may appear narrowed, irregular with hyperechoic walls [19, 20]. Peri-pancreatic fluid collections are less common and not specific for AIP [20]. However, in rare cases, pseudocysts may be seen.

#### Focal AIP

Focal AIP can present as a solitary mass, often seen as a hypoechoic lesion, primarily located in the head of the pancreas. This mass may give the impression of invading nearby vessels and be associated with enlarged peri-pancreatic lymph nodes resembling locally advanced pancreatic cancer (PC) [19, 20]. The main pancreatic duct (MPD) wall may appear thickened, narrowed within the lesion. However, pancreatic duct cut-off sign is not seen [19, 20]. Despite well-established EUS features of AIP reported globally, there is a notable lack of data from Indian centres. In a previous study by Jain et al. [9], 41% patients were diagnosed using endoscopic ultrasound-guided biopsy. This gap highlights the critical need for robust, region-specific studies to validate these findings in the Indian context.

### Other organ involvement

The biliary tree is the most common extra-pancreatic organ involvement in AIP and was found to affect 58% of patients in a Japanese survey [21]. EUS provides a comprehensive view of the entire common bile duct, helping to identify the cause for biliary strictures. A typical EUS feature of the common bile duct is homogeneous, regular thickening of the bile duct wall, known as the “sandwich-pattern”. This pattern is characterized by an echopoor intermediate layer with hyperechoic inner and outer layers (Fig. 3). Along with the bile duct, gallbladder wall thickening can also be seen in AIP [20, 22]. Intra-ductal ultrasonography (IDUS) can also be used during endoscopic retrograde cholangiography to assess biliary stenosis. Naitoh et al. studied IDUS in 23 IgG4-related sclerosing cholangitis (IgG4-SC) patients and found that circular, symmetric wall thickness with smooth margins and a homogeneous intermediate layer were more common in AIP than cholangiocarcinoma. Additionally, wall thickness in non-strictured areas of IgG4-SC was greater than in Cholangiocarcinoma, with a thickness over 0.8 mm suggesting IgG4-SC [23].

The reported frequency of lymphadenopathy in AIP patients varies widely across EUS studies, ranging from 31.2% to 72% [15, 24, 25]. Hilar lymphadenopathy is among the most commonly reported extra-pancreatic findings. Enlarged lymph nodes are also observed in the peri-pancreatic and celiac regions [21]. However, the conventional EUS criteria for predicting malignant lymph nodes such as size (> 1 cm in diameter on the short axis), hypoechoic appearance, round shape and smooth border may not be reliable in pancreato-biliary disorders, making it challenging to distinguish them from those associated with AIP based on EUS features alone [26, 27]. AIP can have vascular invasion of medium and large-sized vessels due to the inflammatory process. Peri-pancreatic vascular involvement is a common finding in AIP, detected in 44% to 67% of cases on CT. The most frequently observed vascular abnormalities include stenosis or occlusion of the splenic vein and/or superior mesenteric-portal vein, along with peri-gastric collateral formation [28–30]. In a study of 14 AIP patients, EUS suspected portal or mesenteric vein invasion in 21% of cases, compared to 14% on CT. Notably, none of these patients developed pancreatic cancer during follow-up [19].

### Staging of the disease

AIP is classified into early-stage disease, which is characterized by a good response to corticosteroid therapy and advanced-stage disease, which is marked by stone and cyst formation resembling findings in chronic alcoholic pancreatitis [31, 32]. EUS can help differentiate between the stages of AIP. Kubota et al. [33] demonstrated that lobularity and a hyperechoic pancreatic duct margin are characteristic EUS features of early AIP, compared to advanced AIP.

### To differentiate from pancreatic cancer

AIP constitutes a significant portion of pancreatic resections performed for suspected malignancy. In two surgical series, AIP accounted for 19.5% and 23.4%, respectively, of all cases where benign disease was diagnosed following pancreatic resection performed for suspected malignancy [34, 35]. In an Indian study, AIP was initially misdiagnosed as pancreatic cancer due to overlapping clinical and imaging features [6]. In another series of IgG4-related gastrointestinal disease, one-third of cases were diagnosed after surgery, most of which were autoimmune pancreatitis [36]. Therefore, correctly diagnosing focal AIP and distinguishing it from PC is crucial, although it is challenging due to overlapping clinical and imaging features. In a study by Guo et al., EUS features for focal AIP included diffuse hypoechogenicity, hyperechoic foci, bile duct wall thickening and peripancreatic hypoechoic margins. In contrast, PC showed focal hypoechogenicity, absence of parenchymal heterogeneity, pancreatic duct dilation and vessel involvement. The model demonstrated excellent diagnostic accuracy, with sensitivity ranging from 83.7% to 91.8% and specificity from 93.3% to 95.6% [25]. Tacelli et al. [37] suggested that thickened pancreatic and bile duct walls, an elastic pattern and the absence of vessel infiltration were found to independently support the diagnosis of focal AIP1 over PC. Along with conventional EUS features, advanced EUS modalities such as EUS elastography and contrast-enhanced EUS (CE-EUS) can aid in diagnosing AIP and differentiating between focal AIP and PC. Table 2 summarizes the findings comparing AIP and pancreatic cancer.

**Table 2:** Differentiating autoimmune pancreatitis from pancreatic cancer using endoscopic ultrasound modalities

Feature	Autoimmune pancreatitis	Pancreatic cancer
**Conventional EUS**		
Pancreatic morphology	Diffuse or focal enlargement with featureless borders; sausage-shaped pancreas	Hypoechoic irregular mass, often focal
Parenchymal echotexture	Hypoechoic, homogenous or mildly heterogeneous	Hypoechoic, heterogeneous
Main pancreatic duct (MPD)	Long segment narrowing with minimal upstream dilation	Abrupt ductal cut-off with significant upstream dilation
Peri-pancreatic rim	Hypoechoic “capsule-like” rim often present	Absent
Lymphadenopathy	Multiple, small, homogenous nodes; commonly non-malignant appearing	Usually few, round, hypoechoic, irregular borders; higher suspicion of malignancy
Vascular invasion	Rare	Common (especially with major vessel encasement or narrowing)
Peri-vascular changes	**Peri**-**vascular hypoechoic cuffing or halo**	Rare
Vessel wall thickening	**Diffuse concentric**	Focal, eccentric
**EUS Elastography**		
Elastography pattern	Homogeneous green pattern (softer tissue)	Predominantly blue pattern (harder tissue)
Strain ratio	Low strain ratio (< 3)	High strain ratio (> 6–10)
**CH-EUS**		
Enhancement pattern	Homogeneous or iso-/hyperenhancement	Hypoenhancement (due to hypo-perfusion of cancerous tissue)

*CH-EUS* contrast-enhanced harmonic EUS, *EUS* endoscopic ultrasound

### EUS elastography

**Fig. 5:**
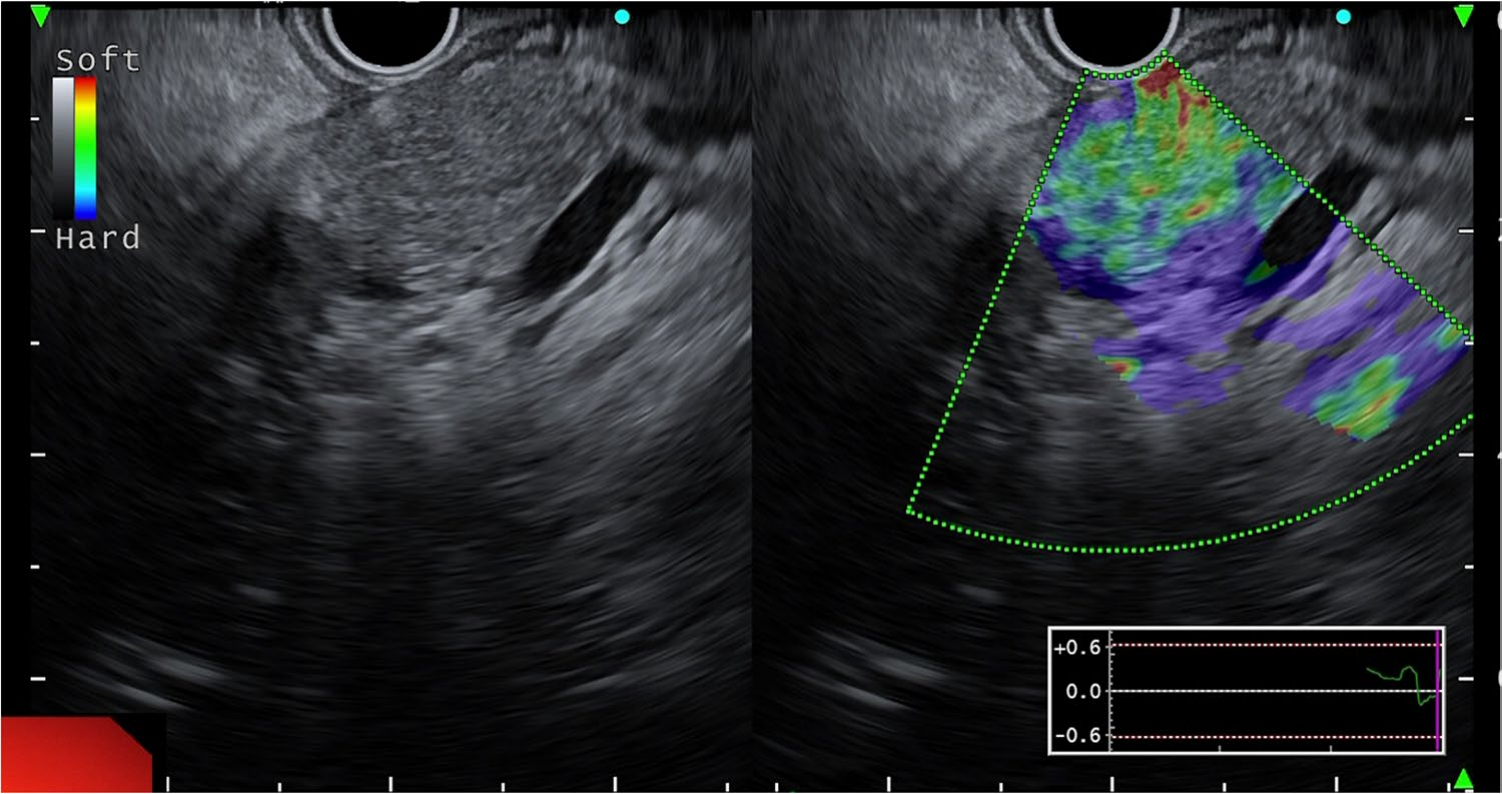
Endoscopic ultrasound showing heterogenous parenchymal echogenicity of the pancreas (left panel); On strain elastography the parenchyma appears diffusely firm (right panel)

EUS elastography assesses the elasticity of pancreatic tissue, with regions of increased stiffness potentially indicating fibrosis, a characteristic feature of AIP. There are two main types of EUS elastography: strain elastography and shear-wave elastography (SWE). Strain elastography assesses tissue stiffness by measuring relative deformation in response to external or internal pressure, displaying results as a color-coded map. In AIP, qualitative elastography shows an inflamed area with a predominantly green pattern and slight red or yellow lines, while normal parenchyma appears uniformly green (Fig. 5) [38]. Elastography may also help differentiate AIP from PC, as AIP shows a diffuse stiffness increase, while PC has localized stiffness [39]. However, it is semi-quantitative and depends on subjective image selection.

In contrast, SWE generates shear waves within the tissue and measures their propagation speed to provide absolute elasticity values, offering a more objective and reproducible assessment. A study evaluated the feasibility and clinical utility of EUS shear-wave elastography (EUS-SWE) in diagnosing and monitoring AIP. Tissue elasticity was measured in 160 patients, including 14 with AIP. EUS-SWE had a 97.6% success rate, with the median shear-wave velocity (Vs) in AIP patients (2.57 m/s) significantly higher than in normal controls (1.89 m/s, *p* = 0.0185). Steroid therapy reduced Vs from 3.32 to 2.46 m/s (*p* = 0.0234). The study concluded that EUS-SWM is a feasible and reliable tool for diagnosing AIP and assessing treatment response [40].

### Contrast-enhanced EUS (CE-EUS)

CE-EUS is an advanced imaging technique that enhances the evaluation of pancreatic lesions by providing real-time visualization of microvasculature and tissue perfusion. Its sub-type, contrast-enhanced harmonic EUS (CH-EUS) offers clear, real-time imaging of blood vessels in the target tissue, without artifacts commonly seen with Doppler mode. Additionally, CH-EUS enables the creation of time-intensity curves (TICs) and graphing of the changes in brightness over time through contrast [41]. A study by Cho et al. assessed the usefulness of CH-EUS in differentiating focal AIP from PC. Among 80 patients, focal AIP showed significantly higher rates of hyper to iso-enhancement (89% vs. 13%), homogeneous contrast distribution (81% vs. 17%) and absence of irregular vessels (85% vs. 30%) compared to PC (all *p* < 0.05). Combining CH-EUS features improved specificity to 94%. These findings suggest CH-EUS is a valuable non-invasive tool for distinguishing f-AIP from PC [42].

In another study by Dong et al. [43], CE-EUS helped differentiate AIP from PC. Among 60 AIP and 16 PC cases, AIP lesions showed iso or hyperenhancement, while PC lesions were mostly hypoenhancing (*p* < 0.01). Similarly, Ishikawa et al. investigated the role of CE-EUS in differentiating AIP from PC. Among 36 AIP and 36 PC patients, key EUS findings included hyperechoic areas in AIP and anechoic areas in PC, with PC exhibiting clearer borders and greater MPD dilation. CE-EUS revealed persistent enhancement in AIP (75%) and rapid contrast washout in PC (75%), achieving 75% sensitivity and 100% specificity. These results highlight the diagnostic value of EUS combined with CE-EUS in distinguishing AIP from PC [44].

A study by Imazu et al. evaluated whether quantitative perfusion analysis using the “Time Intensity Curve” (TIC) with CH-EUS can differentiate AIP from PC. TIC analysis in eight AIP and 22 PC patients showed significantly higher peak intensity (PI) and maximum intensity gain (MIG) in AIP. An MIG cut-off of 12.5 demonstrated high sensitivity and specificity. Thus, CH-EUS with TIC may improve the accuracy of distinguishing AIP from PC [45]. However, TIC analysis can be challenging as it requires precise techniques, specialized software and careful selection of the region of interest (ROI) to ensure accurate results. Another study by Ishikawa et al. evaluated a simplified multiphase CE-EUS method for differentiating pancreatic solid lesions without relying on a time-intensity curve (TIC). Among 210 patients, including 142 with PC, lesion enhancement patterns at 20, 40 and 60 seconds post-contrast were analyzed. Most PC cases showed an early enhancement followed by a hypovascular pattern, achieving 83.1% sensitivity, 86.8% specificity and 84.3% accuracy. Histopathological differences in tumor infiltration and neural invasion were noted. These findings suggest that multiphase CE-EUS is a practical and effective alternative to TIC for diagnosing pancreatic lesions [28].

### EUS-guided tissue sampling

The ICDC outline a multifaceted approach to diagnosing AIP. When imaging findings are characteristic, the diagnosis can often be made without histologic confirmation, allowing for prompt steroid therapy. However, in cases with atypical imaging or a mass-forming lesion, distinguishing AIP from PC becomes essential for determining the appropriate treatment. In such instances, pancreatic biopsy sampling serves as the gold standard for confirming AIP, distinguishing it from cancer and identifying whether it is Type-1 or Type-2 AIP [46].

EUS-guided fine needle aspiration (FNA) and fine needle biopsy (FNB) are commonly used for obtaining pancreatic tissue in suspected AIP, but their diagnostic performance differs. FNA, traditionally used for cytologic evaluation, often yields insufficient tissue for histopathologic assessment, limiting its utility in diagnosing AIP, which relies on architectural features such as storiform fibrosis and obliterative phlebitis. It can only evaluate one of the four diagnostic criteria (IgG4 + cells), which is insufficient for diagnosing AIP [47]. In contrast, FNB provides better-preserved core tissue samples, enhancing the ability to meet histologic criteria for AIP. Meta-analyses and comparative studies suggest that FNB has a significantly higher diagnostic yield than FNA while maintaining comparable safety profiles. Therefore, FNB is increasingly preferred over FNA for obtaining adequate histologic samples in AIP diagnosis (Fig. 5).

Yoon et al. conducted a meta-analysis to compare the efficacy of EUS-guided FNA and FNB in diagnosing AIP. Analyzing data from nine FNA studies (309 patients) and seven FNB studies (131 patients), they found that FNB had a significantly higher pooled diagnostic yield for level 1 or 2 histologic criteria of AIP (87.2% vs. 55.8%, *p* = 0.030), while histologic tissue procurement rates were similar between the two techniques (91.3% for FNA vs. 87.0% for FNB, *p* = 0.501). Adverse events were comparable between groups. Additionally, 19G needles had a superior diagnostic yield compared to 22G needles (88.9% vs. 60.6%, *p* = 0.023). These findings suggest that FNB may offer better diagnostic accuracy for AIP, highlighting the need for a standardized definition of histologic sample adequacy [48].

**Fig. 6:**
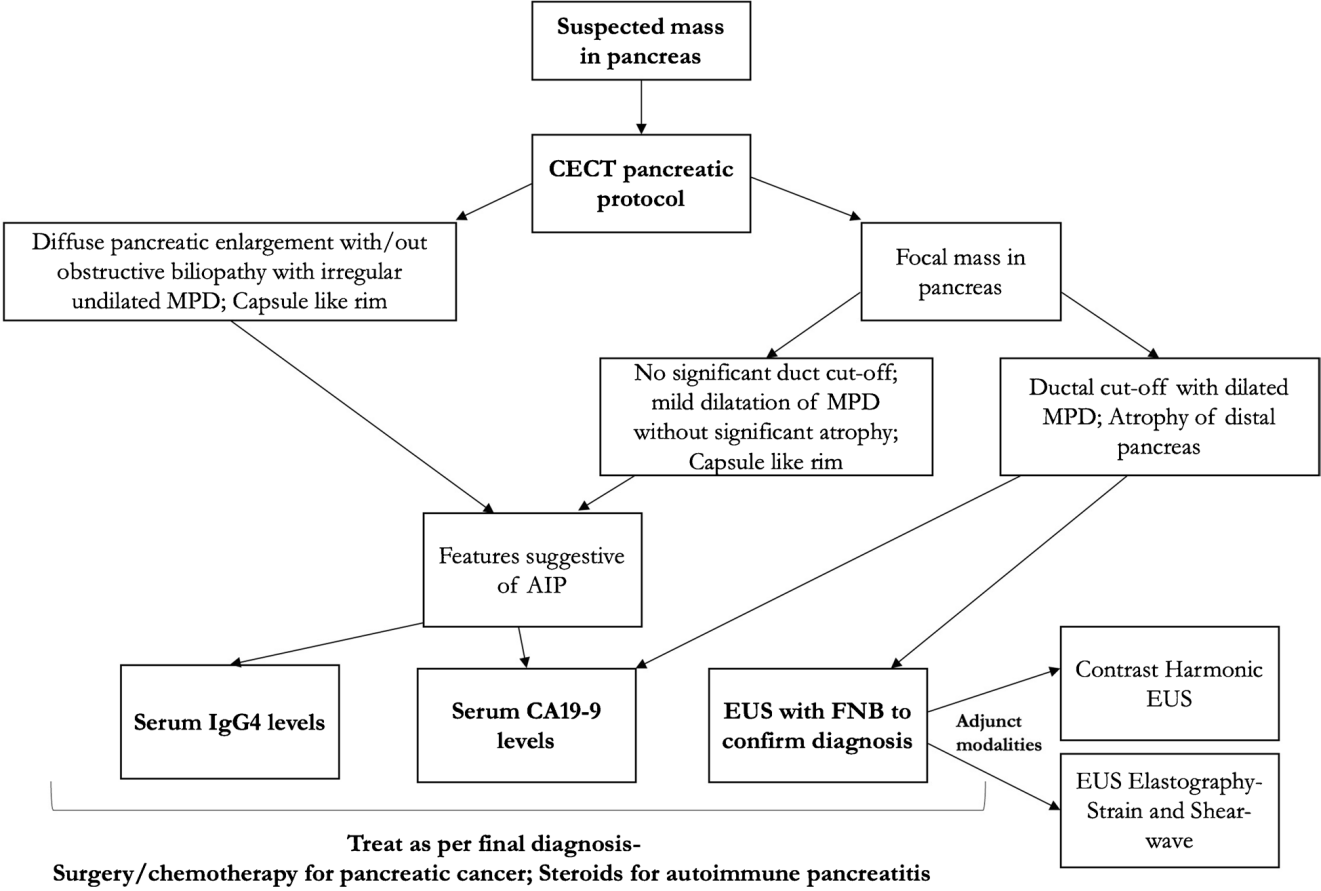
Diagnostic algorithm for suspected mass in pancreas and differentiation from autoimmune pancreatitis. *CECT* contrast-enhanced computed tomography, *MPD* main pancreatic duct, *AIP* autoimmune pancreatitis, *IgG4* immunoglobulin G4, *CA* 19-9 carbohydrate antigen 19-9, *EUS* endoscopic ultrasound, *FNB* fine needle biopsy

A recent meta-analysis by Facciorusso et al. evaluated the diagnostic performance of EUS-guided FNA and FNB in AIP across 15 studies with 631 patients. The overall diagnostic accuracy of EUS tissue acquisition was 54.7%, with FNB significantly outperforming FNA (63% vs. 45.7%, *p* < 0.001). FNB also provided a higher rate of level 1 histological diagnosis (44.2% vs. 21.9%, *p* < 0.001) and definitive histopathology (24.3% vs. 14.7%, *p* < 0.001). The procedure was safe, with less than 1% experiencing post-procedural acute pancreatitis. These findings highlight the modest overall accuracy of EUS-guided sampling in AIP, but support the superior diagnostic yield of FNB over FNA [49]. As a result, EUS-guided tissue acquisition is shifting from FNA to FNB. Initial reports on EUS-FNB utilized Tru-Cut biopsy needles; however, these are no longer available due to limited manoeuvrability. The first second-generation FNB device introduced was a side-fenestrated reverse-beveled needle, but it failed to show significant advantages over standard FNA. More recently, advanced FNB needles with forward-acquiring designs have been developed, including Franseen-tip, fork-tip, Menghini-type and side-fenestrated forward-cutting beveled needles. Studies have demonstrated that these new-generation FNB needles provide superior histologic and diagnostic yields compared to both traditional FNA and earlier reverse-beveled FNB needles for evaluating solid pancreatic lesions. A recent retrospective study by Takada et al. compared the diagnostic performance of three EUS-FNB needles (Franseen needle, Menghini-tip needle and Reverse-bevel needle) for solid pancreatic masses, finding the Franseen needle superior in histologic core procurement, sensitivity and accuracy [50]. Kurita et al. in their prospective, multicentre trial compared two EUS-FNB needles for diagnosing Type-1 AIP. Total 101 patients were evaluated using either a 22-gauge Franseen needle or a 20-gauge forward-bevel needle. The Franseen needle obtained significantly more high-power fields and diagnosed a higher percentage of patients with level 1 or 2 lymphoplasmacytic sclerosing pancreatitis (LPSP) (78% vs. 45%, *p* = 0.001). Given its superior performance, the 22-gauge Franseen needle is recommended for routine histologic diagnosis of Type-1 AIP [51]. While wet suction is said to be better than other suction techniques for tissue sampling of solid pancreatic lesions, there is no data available exclusively for a cohort of AIP [35]. Also since histopathology is a critical issue in diagnosis of AIP, macroscopic on-site examination (MOSE) appears more relevant than rapid on-site examination which usually only gives cytology [52]. Figure 6 summarizes the diagnostic algorithm for differentiating AIP and pancreatic cancer [53]. Ampullary biopsy can aid in diagnosing AIP, especially when serum IgG4 is normal or pancreatic tissue is inaccessible. IgG4-positive staining (> 10 cells/HPF) shows good specificity (89% to 100%) for AIP and the procedure is safe with no major reported complications. The ICDC recommends ampullary biopsy during endoscopic retrograde cholangiopancreatography (ERCP) when AIP is suspected [54].

### Therapeutic monitoring

EUS can effectively visualize steroid-induced changes in the pancreatic parenchyma and ductal structures such as parenchymal hypertrophy, hyperechoic foci, hyperechoic strands, lobularity and a high-echoic margin of the main pancreatic duct. These features often respond to steroid therapy with atrophy seen after therapy (Fig. 5). Unlike abdominal ultrasound, EUS provides clearer imaging of these specific findings, offering a potential advantage in diagnosing and monitoring AIP [24, 55].

With the ongoing advancements in technology, the integration of artificial intelligence (AI) into EUS imaging holds great promise for enhancing diagnostic accuracy. Marya et al. [56] developed an EUS-based convolutional neural network (CNN) to differentiate AIP from PC, chronic pancreatitis (CP) and normal pancreas (NP). Trained on EUS images and videos from 583 patients, the model showed high sensitivity and specificity, including 99% sensitivity and 98% specificity for distinguishing AIP from NP and 90% sensitivity with 93% specificity for AIP vs. PC. This CNN model offers the potential for earlier, more accurate diagnosis of AIP, improving patient outcomes and management [56].

In summary, EUS has become an increasingly valuable tool in the diagnosis and management of AIP, offering detailed imaging and tissue acquisition capabilities. EUS remains an underutilized but pivotal modality in India for the early and accurate diagnosis of AIP. Emerging technologies such as EUS-based artificial neural networks, hold promise for enhancing diagnostic precision but require further validation. To improve consistency in clinical practice, incorporating EUS-specific criteria into existing AIP diagnostic guidelines is essential. Despite these advancements, India lacks multicentre prospective studies—particularly those evaluating the role of EUS in AIP. There is a clear need for collaborative national registries and research networks to generate high-quality, region-specific data and to develop standardized diagnostic algorithms suited to the Indian population.

## Data Availability

Not applicable.

## Declarations

### Disclaimer

The authors are solely responsible for the data and the contents of the paper. In no way, the Honorary Editor-in-Chief, Editorial Board Members, the Indian Society of Gastroenterology or the printer/publishers are responsible for the results/findings and content of this article.

## References

[1] Ghazale TB, Chari ST. Autoimmune pancreatitis. Gastroenterol Clin North Am. 2008;37:277–90. 10.1016/j.gtc.2008.02.004 18499030

[2] Gardner TB, Chari ST. Autoimmune pancreatitis. Gastroenterol Clin North Am. 2008;37:439–60. 18499030 10.1016/j.gtc.2008.02.004

[3] Basyal B, Kc P. Autoimmune pancreatitis. In: StatPearls [Internet]. Treasure Island (FL): StatPearls Publishing. 2025 [cited 2025 February 10]. http://www.ncbi.nlm.nih.gov/books/NBK560769/.

[4] Schneider A, Michaely H, Weiss C, et al. Prevalence and incidence of autoimmune pancreatitis in the population living in the Southwest of Germany. Digestion. 2017;96:187–98. 28957814 10.1159/000479316

[5] Nishimori I, Tamakoshi A, Otsuki M. Prevalence of autoimmune pancreatitis in Japan from a nationwide survey in 2002. J Gastroenterol. 2007;42:6–8. 17520216 10.1007/s00535-007-2043-y

[6] Noor MT, Lal A, Kochhar R, et al. Autoimmune pancreatitis: a report from India. JOP. 2010;11:213–9. 20442514

[7] Chari ST, Smyrk TC, Levy MJ, et al. Diagnosis of autoimmune pancreatitis: the Mayo Clinic experience. Clin Gastroenterol Hepatol. 2006;4:1010–6. 16843735 10.1016/j.cgh.2006.05.017

[8] Rana SS, Gupta R, Nada R, et al. Clinical profile and treatment outcomes in autoimmune pancreatitis: a report from North India. Ann Gastroenterol. 2018;31:506–12. 10.20524/aog.2018.0267 PMC6033754 29991897

[9] Jain AK, Sundaram S, Tyagi U, et al. IgG4-related disorders of the gastrointestinal tract: Experience from a tertiary care centre with systematic review of Indian literature. Indian J Gastroenterol. 2024;43:548–56. 37823986 10.1007/s12664-023-01437-6

[10] Frulloni L, Amodio A, Katsotourchi AM, Vantini I. A practical approach to the diagnosis of autoimmune pancreatitis. World J Gastroenterol. 2011;17:2076–9. 21547125 10.3748/wjg.v17.i16.2076 PMC3084391

[11] Fujii LL, Levy M. EUS in the diagnosis of autoimmune pancreatitis. Pancreapedia [Internet]. [cited 2025 February 9]. https://pancreapedia.org/reviews/eus-in-diagnosis-of-autoimmune-pancreatitis.

[12] Klöppel G, Detlefsen S, Chari ST, Longnecker DS, Zamboni G. Autoimmune pancreatitis: the clinicopathological characteristics of the subtype with granulocytic epithelial lesions. J Gastroenterol. 2010;45:787–93. 20549251 10.1007/s00535-010-0265-x

[13] de Pretis N, Crinò SF, Frulloni L. The role of EUS-guided FNA and FNB in autoimmune pancreatitis. Diagnostics (Basel). 2021;11:1653. 34573995 10.3390/diagnostics11091653 PMC8470670

[14] Overbeek KA, Poulsen JL, Lanzillotta M, et al. Type 1 autoimmune pancreatitis in Europe: clinical profile and response to treatment. Clin Gastroenterol Hepatol. 2024;22:994–1004.e10. 38184096 10.1016/j.cgh.2023.12.010

[15] Zhang SY, Feng YL, Zou L, et al. Endoscopic ultrasound features of autoimmune pancreatitis: the typical findings and chronic pancreatitis changes. World J Gastroenterol. 2021;27:7376–86. 34876796 10.3748/wjg.v27.i42.7376 PMC8611206

[16] Shimosegawa T, Chari ST, Frulloni L, et al. International consensus diagnostic criteria for autoimmune pancreatitis: guidelines of the International Association of Pancreatology. Pancreas. 2011;40:352–8. 21412117 10.1097/MPA.0b013e3182142fd2

[17] Okazaki K, Kawa S, Kamisawa T, et al. Amendment of the Japanese consensus guidelines for autoimmune pancreatitis, 2020. J Gastroenterol. 2022;57:225–45. 35192048 10.1007/s00535-022-01857-9 PMC8938398

[18] Proctor RD, Rofe CJ, Bryant TJC, Hacking CN, Stedman B. Autoimmune pancreatitis: an illustrated guide to diagnosis. Clin Radiol. 2013;68:422–32. 23177083 10.1016/j.crad.2012.08.016

[19] Farrell JJ, Garber J, Sahani D, Brugge WR. EUS findings in patients with autoimmune pancreatitis. Gastrointest Endosc. 2004;60:927–36. 10.1016/s0016-5107(04)02230-8 15605008

[20] Buscarini E, Lisi SD, Arcidiacono PG, et al. Endoscopic ultrasonography findings in autoimmune pancreatitis. World J Gastroenterol. 2011;17:2080–5. 21547126 10.3748/wjg.v17.i16.2080 PMC3084392

[21] Kamisawa T, Okamoto A. Autoimmune pancreatitis: proposal of IgG4-related sclerosing disease. J Gastroenterol. 2006;41:613–25. 16932997 10.1007/s00535-006-1862-6 PMC2780632

[22] De Lisi S, Buscarini E, Arcidiacono PG, et al. Endoscopic ultrasonography findings in autoimmune pancreatitis: be aware of the ambiguous features and look for the pivotal ones. JOP. 2010;11:78–84. 20065561

[23] Naitoh I, Nakazawa T, Ohara H, et al. Endoscopic transpapillary intraductal ultrasonography and biopsy in the diagnosis of IgG4-related sclerosing cholangitis. J Gastroenterol. 2009;44:1147–55. 19636664 10.1007/s00535-009-0108-9

[24] Hoki N, Mizuno N, Sawaki A, et al. Diagnosis of autoimmune pancreatitis using endoscopic ultrasonography. J Gastroenterol. 2009;44:154–9. 19214678 10.1007/s00535-008-2294-2

[25] Guo T, Xu T, Zhang S, et al. The role of EUS in diagnosing focal autoimmune pancreatitis and differentiating it from pancreatic cancer. Endosc Ultrasound. 2021;10:280–7. 34213428 10.4103/EUS-D-20-00212 PMC8411560

[26] Bhutani MS, Hawes RH, Hoffman BJ. A comparison of the accuracy of echo features during endoscopic ultrasound (EUS) and EUS-guided fine-needle aspiration for diagnosis of malignant lymph node invasion. Gastrointest Endosc. 1997;45:474–9. 9199903 10.1016/s0016-5107(97)70176-7

[27] Gleeson FC, Rajan E, Levy MJ, et al. EUS-guided FNA of regional lymph nodes in patients with unresectable hilar cholangiocarcinoma. Gastrointest Endosc. 2008;67:438–43. 18061597 10.1016/j.gie.2007.07.018

[28] Ishikawa T, Hirooka Y, Kawashima H, et al. Multiphase evaluation of contrast-enhanced endoscopic ultrasonography in the diagnosis of pancreatic solid lesions. Pancreatology. 2018;18:291–7. 29449151 10.1016/j.pan.2018.02.002

[29] Matsubayashi H, Uesaka K, Kanemoto H, et al. Reduction of splenic volume by steroid therapy in cases with autoimmune pancreatitis. J Gastroenterol. 2013;48:942–50. 23076542 10.1007/s00535-012-0692-y

[30] Ishikawa T, Itoh A, Kawashima H,, et al. Peripancreatic vascular involvements of autoimmune pancreatitis. J Gastroenterol Hepatol. 2012;27:1790–5. 10.1111/j.1440-1746.2012.07248.x 22849535

[31] Hirano K, Tada M, Isayama H, et al. Long-term prognosis of autoimmune pancreatitis with and without corticosteroid treatment. Gut. 2007;56:1719–24. 17525092 10.1136/gut.2006.115246 PMC2095691

[32] Takayama M, Hamano H, Ochi Y, Saegusa H, Komatsu K, Muraki T, et al. Recurrent attacks of autoimmune pancreatitis result in pancreatic stone formation. Am J Gastroenterol. 2004;99:932–7. 10.1111/j.1572-0241.2004.04162.x 15128363

[33] Kubota K, Iida H, Fujisawa T, et al. Clinical factors predictive of spontaneous remission or relapse in cases of autoimmune pancreatitis. Gastrointest Endosc. 2007;66:1142–51. 18061714 10.1016/j.gie.2007.06.059

[34] Abraham SC, Wilentz RE, Yeo CJ, et al. Pancreaticoduodenectomy (Whipple resections) in patients without malignancy: are they all “chronic pancreatitis”? Am J Surg Pathol. 2003;27:110–20. 12502933 10.1097/00000478-200301000-00012

[35] Weber SM, Cubukcu-Dimopulo O, Palesty JA, et al. Lymphoplasmacytic sclerosing pancreatitis: inflammatory mimic of pancreatic carcinoma. J Gastrointest Surg. 2003;7:129–37; discussion 137-9. 12559194 10.1016/s1091-255x(02)00148-8

[36] Giri S, Afzalpurkar S, Angadi S, Marikanty A, Sundaram S. Comparison of suction techniques for EUS-guided tissue acquisition: systematic review and network meta-analysis of randomized controlled trials. Endosc Int Open. 2023;11:E703–11. 37564335 10.1055/a-2085-3674 PMC10411163

[37] Tacelli M, Zaccari P, Petrone MC, et al. Differential EUS findings in focal type 1 autoimmune pancreatitis and pancreatic cancer: a proof-of-concept study. Endosc Ultrasound. 2022;11:216–22. 35142701 10.4103/EUS-D-21-00111 PMC9258021

[38] Janssen J, Schlörer E, Greiner L. EUS elastography of the pancreas: feasibility and pattern description of the normal pancreas, chronic pancreatitis, and focal pancreatic lesions. Gastrointest Endosc. 2007;65:971–8. 17531630 10.1016/j.gie.2006.12.057

[39] Conti CB, Cereatti F, Drago A, Grassia R. Focal autoimmune pancreatitis: a simple flow chart for a challenging diagnosis. Ultrasound Int Open. 2020;06:E67–75. 10.1055/a-1323-4906 PMC7815440 33490857

[40] Ohno E, Hirooka Y, Kawashima H, et al. Feasibility and usefulness of endoscopic ultrasonography-guided shear-wave measurement for assessment of autoimmune pancreatitis activity: a prospective exploratory study. J Med Ultrason (2001). 2019;46:425-33. 30993580 10.1007/s10396-019-00944-4 PMC6765472

[41] Yokoyama K, Kanno A, Miwata T, et al. Efficacy of contrast-enhanced endoscopic ultrasonography for the diagnosis of pancreatic tumors. Diagnostics. 2022;12:1311. 35741121 10.3390/diagnostics12061311 PMC9222168

[42] Cho MK, Moon SH, Song TJ, et al. Contrast-enhanced endoscopic ultrasound for differentially diagnosing autoimmune pancreatitis and pancreatic cancer. Gut Liver. 2018;12:591–6. 29699060 10.5009/gnl17391 PMC6143455

[43] Dong Y, D’Onofrio M, Hocke M, et al. Autoimmune pancreatitis: imaging features. Endosc Ultrasound. 2018;7:196–203. 28836516 10.4103/eus.eus_23_17 PMC6032703

[44] Ishikawa T, Hirooka Y, Itoh A, et al. Usefulness of endoscopic ultrasonography (plain combined with contrast-enhanced) in the differentiation between autoimmune pancreatitis and pancreatic cancer. Gastrointest Endosc. 2009;69:AB246.

[45] Imazu H, Kanazawa K, Mori N, et al. Novel quantitative perfusion analysis with contrast-enhanced harmonic EUS for differentiation of autoimmune pancreatitis from pancreatic carcinoma. Scand J Gastroenterol. 2012;47:853–60. 22507131 10.3109/00365521.2012.679686

[46] Yonamine K, Koshita S, Kanno Y, et al. Diagnostic value of homogenous delayed enhancement in contrast-enhanced computed tomography images and endoscopic ultrasound-guided tissue acquisition for patients with focal autoimmune pancreatitis. Clin Endosc. 2023;56:510–20. 37032116 10.5946/ce.2022.142 PMC10393566

[47] Majumder S, Chari ST. EUS-guided FNA for diagnosing autoimmune pancreatitis: does it enhance existing consensus criteria? Gastrointest Endosc. 2016;84:805–7. 27742043 10.1016/j.gie.2016.05.046

[48] Yoon SB, Moon SH, Song TJ, Kim JH, Kim MH. Endoscopic ultrasound-guided fine needle aspiration versus biopsy for diagnosis of autoimmune pancreatitis: systematic review and comparative meta-analysis. Dig Endosc. 2021;33:1024–33. 33030283 10.1111/den.13866

[49] Facciorusso A, Barresi L, Cannizzaro R, et al. Diagnostic yield of endoscopic ultrasound-guided tissue acquisition in autoimmune pancreatitis: a systematic review and meta-analysis. Endosc Int Open. 2021;9:E66–75. 33403238 10.1055/a-1293-7279 PMC7775812

[50] Takada K, Yazumi S, Hara K, et al. Comparison between three types of needles for endoscopic ultrasound-guided tissue acquisition of pancreatic solid masses: a multicenter observational study. Sci Rep. 2023;13:4097. 36871105 10.1038/s41598-023-30920-5 PMC9985625

[51] Kurita A, Yasukawa S, Zen Y, et al. Comparison of a 22-gauge Franseen-tip needle with a 20-gauge forward-bevel needle for the diagnosis of type 1 autoimmune pancreatitis: a prospective, randomized, controlled, multicenter study (COMPAS study). Gastrointest Endosc. 2020;91:373–81.e2. 31654634 10.1016/j.gie.2019.10.012

[52] Sundaram S, Chhanchure U, Patil P, et al. Rapid on-site evaluation (ROSE) versus macroscopic on-site evaluation (MOSE) for endoscopic ultrasound-guided sampling of solid pancreatic lesions: a paired comparative analysis using newer-generation fine needle biopsy needles. Ann Gastroenterol. 2023;36:340–6. 37144017 10.20524/aog.2023.0790 PMC10152805

[53] Harindranath S, Sundaram S. Approach to pancreatic head mass in the background of chronic pancreatitis. Diagnostics (Basel). 2023;13:1797. 37238280 10.3390/diagnostics13101797 PMC10217770

[54] Moon SH, Kim MH. The role of endoscopy in the diagnosis of autoimmune pancreatitis. Gastrointest Endosc. 2012;76:645–56. 10.1016/j.gie.2012.04.458 22898422

[55] Okabe Y, Ishida Y, Kaji R, et al. Endoscopic ultrasonographic study of autoimmune pancreatitis and the effect of steroid therapy. J Hepatobiliary Pancreat Sci. 2012;19:266–73. 21671062 10.1007/s00534-011-0392-7

[56] Marya NB, Powers PD, Chari ST, et al. Utilisation of artificial intelligence for the development of an EUS-convolutional neural network model trained to enhance the diagnosis of autoimmune pancreatitis. Gut. 2021;70:1335–44. 33028668 10.1136/gutjnl-2020-322821 PMC13163153

